# Systems Biology of Cancer: A Challenging Expedition for Clinical and Quantitative Biologists

**DOI:** 10.3389/fbioe.2014.00027

**Published:** 2014-08-19

**Authors:** Ilya Korsunsky, Kathleen McGovern, Tom LaGatta, Loes Olde Loohuis, Terri Grosso-Applewhite, Nancy Griffeth, Bud Mishra

**Affiliations:** ^1^Department of Computer Science, Courant Institute, New York University, New York, NY, USA; ^2^Department of Mathematics and Statistics, Hunter College, City University of New York, New York, NY, USA; ^3^Department of Mathematics, Courant Institute, New York University, New York, NY, USA; ^4^Department of Computer Science, The Graduate Center, City University of New York, New York, NY, USA; ^5^Department of Mathematics and Computer Science, Lehman College, City University of New York, New York, NY, USA

**Keywords:** molecular modeling, new algorithms, dynamical models, multi-scale modeling, cancer pathway modeling

## Abstract

A systems-biology approach to complex disease (such as cancer) is now complementing traditional experience-based approaches, which have typically been invasive and expensive. The rapid progress in biomedical knowledge is enabling the targeting of disease with therapies that are precise, proactive, preventive, and personalized. In this paper, we summarize and classify models of systems biology and model checking tools, which have been used to great success in computational biology and related fields. We demonstrate how these models and tools have been used to study some of the twelve biochemical pathways implicated in but not unique to pancreatic cancer, and conclude that the resulting mechanistic models will need to be further enhanced by various abstraction techniques to interpret phenomenological models of cancer progression.

## 1 Introduction

The defeat of cancer was envisioned, somewhat optimistically, after just a few years of research starting with extensive genomic and transcriptomic data collection. Such portrayal of the future might have been inspired by on-going research that has focused on characterizing cancer as a disease of the genome and has galvanized massive data-collection projects, such as the ICGC (International Cancer Genome Consortium) (Zhang et al., 2011) and TCGA (The Cancer Genome Atlas): an atlas “to systematically explore the entire spectrum of genomic changes involved in more than 20 types of human cancer” (TCGA, 2013). Such projects have provided an impetus for developing genomics and bioinformatics tools to study genomic aberrations, driver mutations, loss of heterozygosity, copy number fluctuations, epigenomic modifications, and identifications of classes of oncogenes and tumor-suppressor genes. However, the recent focus has begun to shift to a much more amorphous and dynamic model of cancer, as it has become apparent that a better characterization of the disease must also include the evolution of cancer phenotypes in a heterogeneous population of cells, whose individual types and states need to be understood from single-cell measurements of DNA and RNA, at the very least. The picture of natural somatic evolution of cancer, emerging from recent studies, is quite complex: cancer is driven by numerous pathways, by interactions among multiple heterogeneous subpopulations, the immune system and the microenvironment, and also, by intricate “signaling games” played among cancer stem and progenitor cells, further tempered by metabolic constraints. To treat cancer as a “disease of the phenome,” cancer systems biology research will need to analyze and model complexities of both cell-autonomous and cell-population-level processes.

Consequently, models of cancer evolution may need to deal with state-space trajectories of thousands of rapidly evolving cell-types in a heterogeneous tumor population. The experimental setup to harvest and feed the data to such an algorithm is challenging: it is not yet possible to routinely sample multiple single tumor-cells (either *in situ* or circulating) from a single human patient at multiple stages of their natural progression (unperturbed by any therapy). Our approach may circumvent this problem by using computational systems biology to simulate this progression on phenomenological and mechanistic models.

Primary challenges for cancer systems biologists, as corroborated (Reya et al., 2001; Jordan et al., 2006; Shackleton et al., 2009; Marjanovic et al., 2013) by prominent research biologists, are as follows: (1) The nature and origin of heterogeneity in cancer are not well understood. (2) Cancer stem cells, their interactions with the stroma (normal cells) and the roles they play in the population, especially in choreographing cancer progression are computationally complex and require sophisticated algorithms and modeling techniques. (3) Disentangling how and which cell-autonomous processes manifest at the population level require new analysis tools. Succinctly generating hypotheses and efficiently correlating them to experimental data require highly sophisticated algorithms, which will very likely involve multiple levels of abstraction, composition of qualitative and quantitative models, and symbolic model checking tools that rely on notions of simulation and bisimulation (exact or approximate). These new challenges in modeling and analysis will spur on new research in theoretical computer science. The possible approaches to these challenges are discussed further with illustrative examples.

The paper intends to motivate a disparate group of researchers from multiple disciplines to attack a problem that has not only remained undefeated despite a decades-long all-consuming war against cancer but also has recently revealed new complexities, against which our arsenal has no effective weapons. We wish to inspire game theorists, control engineers, and computer scientists to modify their traditional tools to tame and contain cancer as in many other chronic diseases. We wish to encourage system biologists, bioinformaticists, and oncologists to familiarize themselves with the newer and more powerful tools that rely on abstraction and meta-analysis to overcome the challenges posed by heterogeneity and temporality.

In what follows we focus on the new algorithmic strategies developed to address heterogeneity and temporality as well as other future challenges and obstacles: we start with a summary of classes of models (stochastic, differential, finite-state models, hierarchical, rule-based, and multi-scale) and computational tools (based on execution, simulation, bisimulation, abstraction, composition, and model checking) that are being actively developed by computer scientists. We discuss how these models and tools can be applied to cancer using examples of some of the biochemical pathways implicated in pancreatic cancer (e.g., TGF-β signaling). We also identify critical gaps in the currently available toolkits and future research directions.

The most common form of pancreatic cancer, pancreatic ductal adenocarcinoma (PDAC), is still one of the least understood and most difficult to diagnose and treat of cancers. A central question to ameliorating these difficulties is to identify the genetics drivers behind the origins and progression of PADC. Although PADC is known (Delpu et al., 2011) to arise from 3 different types of precursor lesions, pancreatic intraepithelial neoplasia (PanIN), intraductal papillary mucinous neoplasms (IPMN), and mucinous cystic neoplasms (MCN), the genetic events that characterize the lesions and the transition from lesion to tumor are unknown. It is well accepted that while particular genomic events drive tumorigenesis, it is the change in cellular function caused by that event that is selected for through somatic evolution. Intracellular signaling pathways are common targets of these events. Because of their well understood relations to cellular function, pathways are more consistent and regular markers of tumorigenesis. To better understand which pathways are affected in PDAC, Jones et al. (2008) examined several candidate pathways and found 12 primary ones most common in PDAC tumor samples. In particular, they implicated the pathways associated with apoptosis, DNA damage control, regulation of the G1/S transition in the cell cycle, hedgehog signaling, homophilic cell adhesion, integrin signaling, c-Jun N-terminal kinase signaling, KRAS signaling, regulation of invasion, small GTPase-dependent kinase signaling, TGF-β signaling, and Wnt/Notch signaling. A better understanding of these pathways, how they interact, and how they are affected in PDAC will lead to better clinical diagnosis and intervention.

The rest of the paper is organized as follows. Section 2 summarizes models and tools currently used to represent and analyze dynamical systems in systems biology. Section 3 discusses the need for novel tools to deal with the influx of new personalized data. In Sections 2 and 3, we also turn to several systems biological examples, all related to cancer, which we have explored in the context of a National Science Foundation Expedition-in-Computing project. Our team focused and developed systems for model checking, robustness analysis, multi-scale analysis, etc., which have played a strong role in improving our understanding of the pancreatic cancer phenotypes. Our starting point was with the twelve pathways identified by Jones et al. (2008), described above. We describe a few examples using these pathways to motivate the use of the new modeling and analytical tools described above and the additional use of techniques and tools for abstracting, combining, and otherwise manipulating models. We discuss the biological significance of each example, followed by a brief explanation of the results obtained from the application of the chosen tool. Lastly, Section 4 concludes with a discussion on how the new class of tools we propose will affect biological modeling and clinical practice in cancer.

## 2 Models and Tools for Cell-Autonomous Dynamic Processes

Despite their apparent variety, all computational models of dynamic systems are just abstract, succinct, and formal representations of reality; their form almost always consists of two components: state, which describes the most relevant parts of the configuration of the system at some time, and flow, which describes how the configuration will change in the near future. Usually, we will prefer models with succinct state-space description, but only to the extent that this need for succinctness does not introduce unacceptable distortion in the dynamic behavior of the model. Within a framework comprising such models, researchers have developed powerful tools to compare, translate, and combine formal models of disparate types. Two important weapons in a systems biologist’s arsenal are the processes of abstraction and composition: abstraction facilitates translations among representations, as needed, while composition enables construction of complex, multi-scale, and systems-level models built from simpler component structures.

Analytical tools comprise the other half of the toolkit. They allow for the examination of model properties beyond basic simulation. However, tool applicability is inherently limited by the fact that a specific tool might have been developed originally for use in one specific class of models. Table 1A provides a sense of the compatibility of some key analytical tools for a broad variety of model classes. To construct this table, we relied on an extensive literature survey of each model class and tool (White, 1977; Dytham, 1995; Bengtsson et al., 1996; Henzinger et al., 1997; Cozman, 1997; Ghosh and Tomlin, 2001; Alur et al., 2001; Bandini et al., 2001; Barton and Lee, 2002; Sutner, 2002, 2009; Wang et al., 2002; Antoniotti et al., 2003a,b; Shmulevich et al., 2003; Ghosh et al., 2003; Friedman and Koller, 2003; Lincoln and Tiwari, 2004; Janes et al., 2004; Friedman, 2004; Li and Chan, 2004; Kwiatkowska et al., 2004; Ihekwaba et al., 2004; Hagiya et al., 2004; Das et al., 2004; Pe’er, 2005; Fauré et al., 2006; Reeves et al., 2006; Langmead et al., 2006; Kim et al., 2006; Chaouiya, 2007; Saez-Rodriguez et al., 2007; Fränzle and Herde, 2007; Narasimhan and Biswas, 2007; Wilkinson, 2007; Sandmann, 2007; Mukherjee and Speed, 2008; Clarke et al., 2008; Sandmann and Wolf, 2008; Ryu et al., 2008; Donaldson and Gilbert, 2008; Figueirêdo et al., 2008; Yuceer et al., 2008; Qian and Dougherty, 2009; Tatyana et al., 2009; Wartlick et al., 2009; Sobie, 2009; Langmead, 2009; Didier et al., 2009; Müssel et al., 2010; Sarkar and Sobie, 2010; Campagna and Piazza, 2010; Bortolussi and Policriti, 2010; Garmaroudi et al., 2010; Yang and Lin, 2010; Donzé et al., 2010; Gunawardena, 2010; Vikram et al., 2010; Kobayashi and Hiraishi, 2010, 2011; Gong et al., 2010, 2011a,b,c; Dimitrova et al., 2011; Grosu et al., 2011; Alfieri et al., 2011; Fischer and Kaiser, 2011; Aldinucci et al., 2011; Brim et al., 2011; Sarkar et al., 2012; Horvath, 2012; Iyengar et al., 2012). In this table, each row represents a class of models. From top to bottom, the models range over Bayesian networks, Boolean networks, ordinary differential equations (ODEs), stochastic models, Petri nets, hybrid automata, cellular automata, and partial differential equations (PDEs), each with differing notions of states (discrete, continuous, hybrid, etc.) and flows (transition, evolution, dynamics, etc.). In addition, we chose these models to represent a broad range of model features, including deterministic, non-deterministic, spatial, non-spatial, continuous, discrete, temporal, and logical. Each column, on the other hand, represents a tool. From left to right (in order of increasing complexity), they encompass: parameter estimation, sensitivity analysis, reachability analysis, and model checking of properties describable in propositional temporal logic.

**Table 1:** **Tools tables**. **(A)** A table of references for the use of each analytical tool in each model type, where available. The colors denote availability, as specified in the legend. **(B)** A (non-exhaustive) table of available implementations of analytical tools described in the previous sections.

**(A)**					

	Parameter estimation	Sensitivity analysis	Robustness	Reachability	Model checking
Bayesian networks					
Boolean networks					
Ordinary differential					
Equations					
Stochastic models					
Petri nets					
Hybrid automata					
Cellular automata					
Partial differential					
Equations					

**(B)**					

**Analytical tool**		**Resource**			

Parameter estimation		Simbiology			
		JSim, Polynome, and PyMorph			
		PARES			
Sensitivity analysis		MATLAB – systems biology toolbox			
		Simbiology			
Robustness analysis		MATLAB – systems biology toolbox			
		R sensitivity package			
		BIOCHAM			
Reachability analysis		MATLAB – robust control toolbox			
		PROD, TReX, and RAMAS			
Model checking		SMV, HyTECH, and HySAT			
		UPPAAL, PRISM, and NuSMV			

*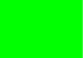 Available*.

*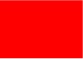 Unavailable*.

*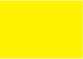 Partially available*.

Each entry represents the availability of the tool for the model class. Red implies that the tool is unavailable or inapplicable. Yellow denotes limited applicability. Green denotes wide-spread applicability across models in that class. For obvious reasons, the simpler tools generally have a wider range of applicability than do the complex ones. The most complex tools have proven difficult to adapt to novel circumstances, thus motivating the use of abstraction to broaden their range of applicability. Traditionally, model abstraction has been used to create models that are structurally simpler, but that have the advantage of facilitating rapid analysis by efficient algorithms and provide easily comprehensible explanations of properties and counter-examples. Table 1B provides examples of implementations of these tools.

### 2.1 Examples

Abstraction provides simplification. For examples, models and analyses that can be constructed and performed, we briefly summarize the findings of two studies on some of the 12 pathways implicated in pancreatic cancer. More details are included in Sections 2.1.1 and 2.1.2, and for more information on the tools used, see Section 2.2.

The first study (Gong et al., 2011c) uses a Boolean circuit as an abstraction of several interacting pathways, including MDM2, P53, NFκB, and HMGB1, and performs symbolic model checking on the resulting abstract circuit. Among many findings, it confirmed, as expected, that P53 can induce the transcription of MDM2, while MDM2 is a negative regulator of P53, and that NFκB’s activation is not a necessary checkpoint that the cancer cell must go through to achieve both proliferation and immortality. Other local analyses related to such abstraction involve: reachability analysis, local and global robustness analysis, parameter identification, and analysis of their sensitivity, etc.

The second study investigates a published model of extrinsically induced apoptosis (Albeck et al., 2008) using parameter sensitivity analysis based on a popular statistical tool called partial least squares regression. The analysis reveals 6 enzymatic reactions that contribute substantially to the time it takes the cell to commit to apoptosis from the initial ligand binding event. Interestingly, all 6 reactions occur prior to the permeabilization of the membrane, confirming the accepted theory that permeabilization is the non-reversible step that commits the cell to apoptosis.

#### 2.1.1 Model checking Boolean model of pancreatic cancer pathways

While there is a plethora of chemical reagents in a cell, which, in principle, can react with one another, most of these reactions do not happen under normal physiological conditions (temperature, pH, etc.). Instead, they are tightly regulated by reaction-specific enzymes and the genes that code for them. Thus, gene regulatory networks are characterized by sharp transitions, in which some subset of reactions is turned on, while the others turned off. Thomas et al. have used Boolean models to describe and analyze this behavior of gene regulatory networks (Thomas, 1991, 1998; Bornholdt, 2008), and have shown that it can be well approximated by asynchronous Boolean networks, in which genes are represented as nodes and the regulation by wiring.

**Figure 1:**
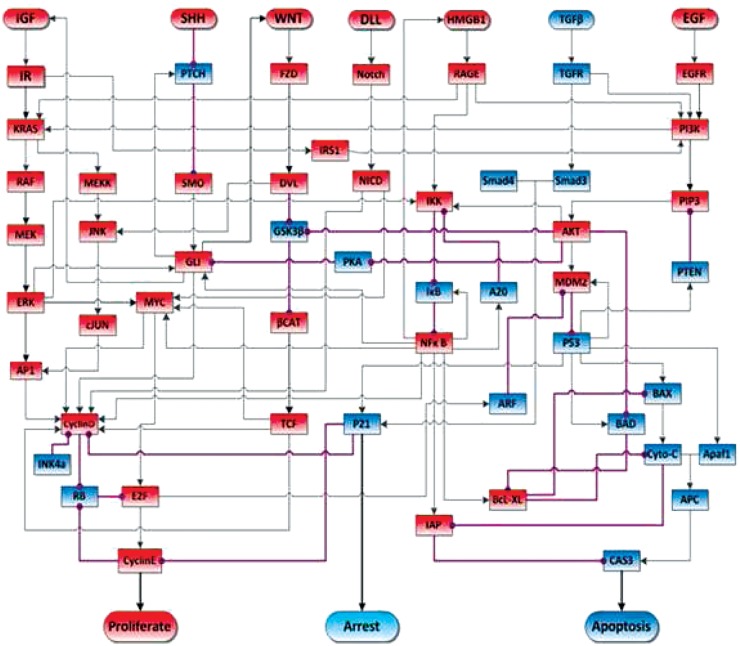
**Schematic view of signal transduction in the pancreatic cancer model**. Blue nodes represent tumor-suppressor proteins, red nodes represent oncoproteins/lipids. Arrow represents protein activation, circle-headed arrow represents deactivation. The acronyms in each rectangular node stand for signal transduction proteins. The rounded rectangular nodes on the top of the figure stand for ligands that activate the pathways. Finally, the rounded nodes at the bottom stand for a cell behavior activated by the connected effector proteins. Figure adapted from Gong et al. (2011c).

A recent study used model checking of a Boolean model of the HMGB1 pathway to verify several experimentally observed behaviors of cancer cells and to suggest further hypotheses for experimental study (Gong et al., 2011c). Figure 1 shows a circuit diagram representation of this Boolean network. One analytical result was that over-expression of HMGB1 would increase proliferation and decrease apoptosis. This has been experimentally observed, as reported in Kang et al. (2009). Another analytical result is that once the protein Cyclin E is activated by the HMGB1 pathway, and DNA synthesis has commenced, the cell will continue to proliferate, and thus be relatively independent of external controls. This has been identified by Weinberg and Hanahan (2000) as one of the hallmarks of cancer. Another analytical result, which NFκB oscillates after release of HMGB1, had been observed by Hoffmann et al. (2002). Some additional analytical results suggest that P53 can induce the transcription of MDM2, while MDM2 is a negative regulator of P53, and that NFκB’s activation is not a necessary checkpoint that the cancer cell should go through on the path to proliferation and immortality.

These results show that model checking can be a powerful tool for the understanding of biological behaviors, just as it has been a powerful tool for understanding complex electronic circuits. Over the past three decades, as the complexity of the engineered circuits have approached that of the natural biological systems, the engineering community had to develop design automation tools built upon powerful algorithms for circuit validation and model checking, first introduced by Clarke and Mishra (1984). Model checking has now become standard protocol for validating electronic circuits.

#### 2.1.2 Sensitivity analysis of TRAIL-induced apoptosis ODE model

**Figure 2:**
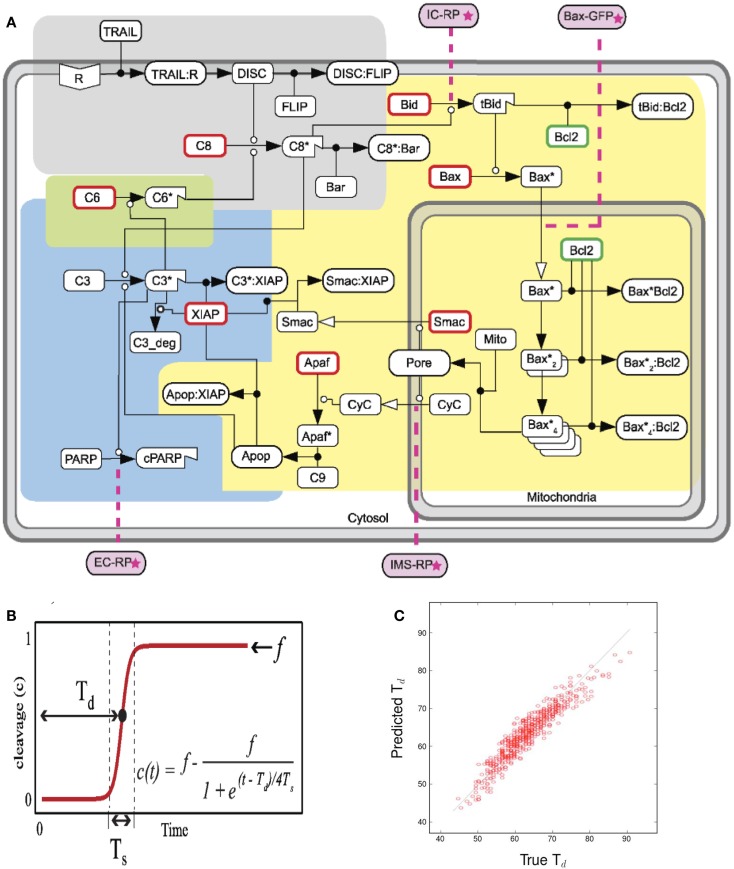
**Figures accompanying the sensitivity analysis**. **(A)** Schematic of the extrinsic apoptosis reaction model. Each color represents a functional pathway. Adapted from Albeck et al. (2008). **(B)** Cleavage of PARP to cPARP in response to TRAIL induction. Adapted from Albeck et al. (2008). **(C)** Predicted vs. True *T_d_*. Quality of linear regression measured by *R*^2^ value.

A partial least squares regression (PLSR) on a well established model for TRAIL-induced apoptosis (Albeck et al., 2008) established the key reactions responsible for the time it takes for the effector protein cPARP to attain its half saturation. Figure 2A represents the reaction network. Each reaction is depicted in the compartment (membrane, cytoplasm, or mitochondria) in which it takes place.

Programed cell death, or apoptosis, is crucial in the development and maintenance of a multi-cellular organism, but also provides a critical ingredient to the character of cancer progression, its dominant phenotypes and heterogeneity. Once certain apoptotic proteins are triggered in a cell, whether from intrinsic or environmental signals, a normal cell commits to a program that results in its eventual cell death. Changes in the cell’s ability to respond to apoptotic signals and the timing behind its response can cause major disturbances in cellular population homeostasis. It is important to understand how robust this response is to genetic mutations.

For this purpose, we analyzed extrinsic apoptosis signal transduction pathway models using tools designed for sensitivity analysis, to identify the key proteins that may be rate-limiting. Rate-limiting proteins are postulated to have the greatest effect on the apoptotic response, and thus suggest important mutations responsible for diseases in which this response is diminished.

In the ODE model, cleavage of the effector protein PARP into cPARP is the indicator of apoptosis. *T_d_*, the time from ligand-receptor binding to the point at which half of all PARP is cleaved, represents the response time of the cell to the apoptosis-inducing signal. Figure 2B shows the typical dynamics of PARP cleavage as well as how to estimate *T_d_*.

Our sensitivity analysis of *T_d_* to all the kinetic rate parameters is based on a linear regression that illustrates the promise of this approach, as indicated by the regression results in Figure 2C.

The reactions with the most impact on *T_d_* are described in Table 2. We found that the most important reactions are those that precede the permeabilization of the mitochondrial membrane. These results suggest that the flood of mitochondrial proteins into the cytoplasm is difficult to control, and that the most effective drugs would target reactions upstream in the cascade.

**Table 2:** **Reactions discovered to affect *T_d_* the most in sensitivity analysis**.

Reaction	Description
$L+R\underset{k_{-1}}{\overset{k_{1}}{⇌}}\;L:R\overset{\kappa_{1}}{\to }\;R^{*}$	Ligand-receptor binding and unbinding and receptor activation
$R^{*}+C8\overset{k_{3}}{\to }\;R^{*}:C8$	Caspase-8 binding to active receptor
$C8^{*}+Bar\overset{k_{4}}{\to }\;C8^{*}:Bar$	Caspase-8 binding to Bar
$C8^{*}+Bid\overset{k_{10}}{\to }\;C8^{*}:Bid$	Caspase-8 binding to Bid
$Bid+Bcl2\underset{k_{-11}}{\overset{k_{11}}{⇌}}\;Bid:Bcl2$	Bid binding and unbinding to Bcl2
$tBid+Bax\overset{k_{12}}{\to }\;tBid:Bax$	Activated Bid binding to Bax

### 2.2 Review of first generation tools

This section includes a brief description of types of models that have been used to simulate dynamic systems in biology as well as the types of tools that have been used to analyze these models.

#### 2.2.1 Model descriptions

##### 2.2.1.1 Rule-based model

Rule-based models provide a concise way to specify highly complex, parameterized interaction networks between agents (e.g., molecules). The user needs only to encode the possible behaviors of complex molecules and the modeling software automatically generates an ODE (Ordinary Differential Equations) or CTMC (Continuous Time Markov Chains) model to simulate directly. Agent interactions can be aggregated into macroscopic behaviors, capturing temporal changes to statistical properties only, and abstracting away the details of the rules.

##### 2.2.1.2 Dynamic Bayesian network

These models represent the joint distribution of all variables in the system over time (a global time). The network (represented graphically) arises from a factorization of this joint distribution into conditional distributions through the application of Bayes’ rule. An edge in the network graph represents a conditional dependence between two variables. Conditional dependences may change over time, so that this is a time-varying graph.

##### 2.2.1.3 Boolean network

This model is characterized by the fact that each variable can only take one of two values, usually on/off or high/low. Boolean networks are commonly used to model gene regulatory networks, in which genes are considered on or off at any given time.

##### 2.2.1.4 Ordinary differential equations (ODEs)

Each variable in this model is characterized by an ordinary differential equation that describes how its rate of production and decay are governed by the concentrations of the ensemble of molecules. Such a system of equations is particularly useful for modeling a large biochemical reaction system, in which the average concentrations of each molecule type can be described through mass action dynamics.

##### 2.2.1.5 Continuous time Markov chain (CTMC)

This class of stochastic models considers objects as stochastic Markov processes, in which state changes are probabilistic rather than deterministic. Markov processes have no memory, that is, the probability of any given state change depends only on the current state, and not on the history of the states.

These models are useful for capturing the dynamics of small reactive systems, in which small stochastic fluctuations have large effects.

##### 2.2.1.6 Petri network

Historically, Petri Nets (PNs) were developed to model chemical reactions, but have been used extensively to reason about resource sharing in concurrent systems (in computer science). Thus, as they are capable of describing variables and consumption/production transformations among variables in terms of a simple bipartite graph, they have been used in describing biological processes involving small number of molecules. This basic formulation has been further extended to include various features that arise in systems biology, such as continuous and hybrid dynamics, stochastic fluctuations, and a notion of real time.

##### 2.2.1.7 Hybrid automata

In a hybrid automata model, system dynamics are continuous in the short term but in the longer term may switch between discrete modes. Hybrid automata can also simplify a system of complex non-linear equations into several simpler interacting components.

##### 2.2.1.8 Cellular automata

Cellular automata (CA) are spatially and temporally discrete models, whose dynamics are controlled by a set of rules, based on the state of the site and those in its neighborhood. Cellular automata are especially useful in modeling spatial processes such as morphological evolution of tumor growth or cell migration.

##### 2.2.1.9 Partial differential equations

PDEs are a widely studied topic in mathematics and generally describe the continuous dynamics of some variables with respect to 2 or more other variables. In systems biology, PDEs are most commonly used to model system dynamics over time and space. They are thus useful for the same kinds of systems as cellular automata.

#### 2.2.2 Tool descriptions

##### 2.2.2.1 Parameter estimation

The dynamical behavior of a model is dependent on all parameter choices, incorporating numerical parameters to topological ones (e.g., the structure of a network).

Experimental measurements are frequently unavailable for important parameters of a model, and expensive to obtain. Parameter estimation tools are available for all types of models to approximate parameters correctly in model construction. Two general approaches to this tool have emerged. One is based on matching model behavior to numerical data, and the other is based on matching it to higher level descriptions in temporal logic. Parameter estimation often results in a range of possible parameter values that allow the model to reproduce the desired specifications. The width of these ranges depends on sensitivity and robustness.

##### 2.2.2.2 Sensitivity analysis

Parameter sensitivity is the degree to which small changes in a parameter’s value affect the overall model behavior. Sensitivity analysis assigns a numerical sensitivity score to each parameter. In a molecular interaction network, these scores yield insight into the relative importance of some molecules in function of the circuit. For instance, a high sensitivity of cell growth to a particular protein may suggest the protein’s roll as an oncoprotein.

##### 2.2.2.3 Robustness analysis

In contrast to the sensitivity analysis, robustness probes the system with large perturbations in the parameter values. Instead of identifying the role of key parameters in the model behavior, robustness tests the conditions under which the model reliably produces the same output. This insight is crucial in drug discovery, in that it identifies the targets needed to alter the model’s output to produce a significantly different behavior.

##### 2.2.2.4 Reachability analysis

The combinatorial state space of a model can be enormous and each of these states can have different biological significance. Reachability analysis aims to quantify the states that are reachable via an execution of the model, given an initial set of conditions. This analysis stems from graph theory, in which the states of a system are modeled as discrete nodes and the dynamics as edge transitions between the nodes. Therefore, for continuous models, a pre-processing discretization step is necessary to transform it into a discrete model. Biologically, this tool is very powerful at predicting the ability of a cell model to reach unfavorable phenotypes. However, a major challenge is to define states that are biologically meaningful.

##### 2.2.2.5 Model checking

Model checking concisely characterizes all possible behaviors of the model with properties in a high-level, expressive language called temporal logic. Such properties include cycles, temporal precedence, and steady state. Like reachability analysis, model checking performs an exhaustive search on the state space of a model, and therefore, relies on a discretization of the state space. Traditional model checking is geared toward efficiently searching large, finite graphs with deterministic transitions. However, biological systems introduce stochastic complex networks, which we model using infinite graphs and probabilistic transitions. To deal with these new challenges, model checking has been recently expanded to include time-bounds to analyze infinite graphs and probabilities and statistical sampling to analyze graphs with probabilistic transitions. The statistical sampling used in model checking employs Monte Carlo sampling, which is a family of algorithms to efficiently sample from a probability distribution that is usually difficult to sample directly.

##### 2.2.2.6 Causal analysis

Large quantitative models offer a rich representation of the dynamics of a system. However, within all the details of the model, it may be difficult to derive a qualitative understanding of a particular event. For instance, a model of intracellular signaling in cancer may include multiple intersecting pathways and thousands of reagents, but it may not be clear, which reagents and reactions are responsible for the activation of NFκB.

Structural causal analysis has emerged in several fields as a way to answer such qualitative questions in systems whose dynamics consist of discrete events (Nielsen et al., 1981; Danos et al., 2007, 2012; Paulevé et al., 2013). In this analysis, the user identifies a particular outcome of interest and the analysis infers the sequence of events leading up to that outcome or a set of events without which the outcome would not occur. Given these sequences or sets of events, the user can focus on those parts of the model that include the relevant events. For instance, we may be interested in which pathway activations led to the transcription of a particular gene. Causal analysis can identify, which pathways directly led to the transcription in the model, even if the user has no initial hypothesis.

The interpretations of causality discussed here are specific to the systems in which they are implemented. Other notions of causality plays a vital role in systems biology and related fields of machine learning (Pearl, 2000; Kleinberg and Hripcsak, 2011) and statistical inference (Loes et al., 2013), with its roots deep in the philosophical foundations of science (Hume, 1902; Cartwright, 2004). For the sake of limiting the scope, we refrain from delving deeper into the various notions and applications of causality.

##### 2.2.2.7 Model reduction

Model reduction (MR) simplifies a model in such a way that the model is more tractable to represent and execute and less prone to overfitting from too many parameters, while the relevant dynamics of the model remain unperturbed. For demonstration, we consider two examples. The first (Feret et al., 2009; Danos et al., 2010) considers a rule-based model of intracellular signaling that would produce an intractably large system of ODEs with the typical semantics. Instead, the authors compute a set of coarse grained variables, called fragments, from the original set of all possible reagents, according to the interactions between the rules. Unlike that of the original set of molecular species, the system of ODEs for these fragments is compact and tractable. In a particular implementation of their method, the authors compute the fragments for a large model of EGFR signaling that consists of 71 rules and 18,051,984,143,555,729,567 molecular species. The model reduction results in only 175,988 fragments, making it possible to construct a feasible system of ODEs to compute the dynamics of these rules. Moreover, the reduction is proven correct (Danos et al., 2010), in that it does not change the quantitative dynamics of the original model.

The second work (Radulescu et al., 2012) focuses on the use of tropical geometry (TG) for model reduction of networks of biochemical reactions, as represented by a system of differential equations. TG has been used in modeling Algebraic Differential Equations that often appear in the study of normal and aberrant biochemical pathways. TG can informally be described as a piece-wise linear or skeletonized version of algebraic geometry, which has been widely applied in enumerative algebraic geometry in the past and more recently, in computational systems biology for model reduction. Thus, TG’s most prominent applications are in obtaining “good” time scale separation in a biochemical reaction network. Its applications are ideal when in the dynamics of certain species, there is a dominant reaction whose effect overshadows that of the rest – not uncommon in an enzymatic reaction. In such a situation, TG can approximate the dynamics of a particular species by only its dominant reaction, until that dominant reaction changes. Tropicalization exploits this idea by simplifying the polynomials that define the rates in the ODE system. Namely, it turns the polynomial into a sum through a log transform and then chooses the largest term by transforming the sum into a max operator. This step reduces the polynomial to a piece-wise smooth function, with fewer parameters but almost identical behavior.

## 3 Models and Tools for Heterogeneous Population Dynamics

### 3.1 Abstraction of ODE model to timed automaton

To reiterate, model abstraction is a process that simplifies a model in such a way that preserves almost all properties that need to be examined. Such simplifications at multiple scales may play a critical role in modeling a heterogeneous population of cells in a tumor.

**Figure 3:**
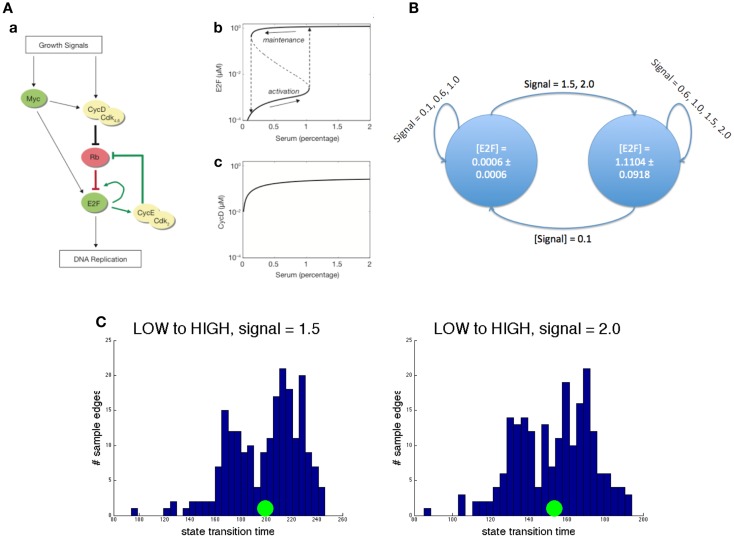
**Abstraction example**. **(A)** A concrete ODE model. Left: circuit diagram. Pointed arrows denote activation while flat head arrows denote inhibition. Right: demonstration of hysteresis in E2F concentration and the lack of it in cyclin D concentration. **(B)** Its reduced abstraction model. Each state is actually characterized by statistics on all species in the original model. Here, the mean and SD of E2F is used for brevity. Each edge is marked by the growth signal values (mean and SD) that cause that transition. **(C)** Two representative edge distributions. The left panel shows the distribution of transition time from the low E2F state to the high one when the growth signal value is 1.5. The right panel shows the same transition for a growth signal value of 2.0. In both, the green circle represents the mean. Notice that the distributions look similar but the mean transition time decreases substantially for the higher input.

We illustrate this approach with an example, highly relevant to cancer: we abstract an ODE model of a bistable switch that controls the G1/S transition in the cell cycle. The key molecules and their interaction leads to a high-level description of the ODE model as portrayed in Figure 3A. The two positive feedback loops governing their interactions lead to two stable states and hysteresis in the transitions between the states. The latter property blocks the circuit from transitioning to the G1 phase once it is in the S phase. The value of the growth signals ranges from 0 (no growth signal) to 2 (full saturation).

Our goals in constructing this abstract model are to identify the steady states of the model, as these are likely to be biologically significant, and to characterize the types of transitions among them. In this example, the resulting abstract model shown in Figure 3B is a two-state model that captures the two steady states of the detailed, mechanistic model. The transition paths are described by a distribution over the time taken by a transition between a pair of states and by the concentration that modulates a certain transition. For instance, in the presence of a high concentration of input signal, the transition from G1 to S phase is marked by a timing distribution centered on a smaller time (see Figure 3C). Notice that both the bistability and hysteresis, the two most important properties of the mechanistic model, are preserved in the abstract model. On the other hand, the exact concentrations of all the molecules in the system are abstracted away. The construction of this simple model was achieved through iterative sampling and simulation, but more complex models may require more advanced techniques, such as those studied in transition state theory and transition path theory (Vanden-Eijnden, 2006).

We performed the abstraction by statistically sampling traces of the concrete ODE model. Each trace began at a stable state perturbed by changing the growth signal and ended when the reagent concentrations reached steady state again. From this procedure, the result of each trace was a starting concrete state, an ending concrete state, and the transition time to get from one to the other. The abstract states were identified by performing k-means clustering on all the starting and ending states of the trace samples, with increasing numbers of clusters. We chose the result that produced the smallest variance of reagent concentrations within clusters, while minimizing the number of clusters. To compute the transition times between some abstract states A and B, for instance, we first labeled the beginning and ending state of each trace sample with the closest abstract states, respectively. Then we considered all traces that started in abstract state A and ended in abstract state B, at a particular growth signal value, and used the transition times of these samples to compute statistics^1^ on the transition time between abstract states A and B, at the same growth signal value.

The gain we have achieved by abstracting a simple ODE model into a simpler discrete state model may not be clear in the context of analyzing just a single cell. However, assured that the abstraction is correct, we can use the approximate dynamics to model each cell in a population of a very large number of cells. Note that such an analysis for large mechanistic models would be intractable for realistic cell populations. Instead of modeling the detailed biochemical interactions within a cell, we view each cell as a strategic agent, interacting stochastically with other cells and its own microenvironment. This game theoretic perspective may illuminate emergent behaviors of the population that were impossible to observe in the single-cell simulations.

This simple example raises many questions about the nature of models, their relationships to one another, and the possibility of constructing composite models out of modular ones. While we observed that the abstract model above captures two key dynamical properties of the original model, are there guarantees about other dynamical information that we may have lost? For instance, was there a rare but important third state that could produce large population-level effects? It is imperative to formally describe the similarity and distance between these two models, which ostensibly represent the same biological system. Finally, how exactly would we construct a composite model from these abstract models to capture their biochemical and mechanical interactions, which are not specified in the single-cell models?

#### 3.1.1 Formal definition of the abstract model

In this section, we provide a formal definition of the model. This section is meant for readers with a computational background who are interested in the formal details of the model. Reader can safely omit this section and refer instead to the informal description given earlier in the paper.

The formal definition of the timed discrete state abstract model follows.

$$\begin{array}{llll} & \text{Model}\;M=<S,E,I>\; & & \;\\ & \text{Abstract States}\;S=\left(s_{1},s_{2},\;\ldots \;s_{n}\right)\;\; & & \;\\ & \text{Concrete State}\;s=\left(p_{1},p_{2},\;\ldots \;p_{k}\right),p_{i}\in R^{nspecies}\; & & \;\\ & \text{Edges}\;E=S\times S\times I\to \Delta \left(R\right)\; & & \;\\ & \text{Input Values}\;I=\left(in_{1},in_{2},\;\ldots \;in_{ninput}\right),in_{i}\in R\; & & \; \end{array}$$

The model is a 3-tuple of a set of abstract states, a set of input values, and a set of edges. Each abstract state is currently characterized by a set of clustered concrete model states, although in the future, abstract states would be more succinctly described using some distribution over the ODE network state. An edge is a map from one abstract state (i.e., start state), another abstract state (i.e., end state), and an input (e.g., extracellular signal) to a probability function over the time. Simply, it estimates the time it takes to get from state 1 to state 2 given some input. The set of inputs is the set of all possible inputs to the network, as described in the edge definition.

### 3.2 Composition of liver model with agent based population model

This section illustrates how composition of models allows exploration of the interaction between two or more disparate systems. The goal of the study described here was to determine the optimal dosing schedule for treatment with Taxol, which is a chemo-therapeutic drug against many forms of cancer. The main result was an optimal schedule, which would avoid liver damage while eliminating cancer cells.

**Figure 4:**
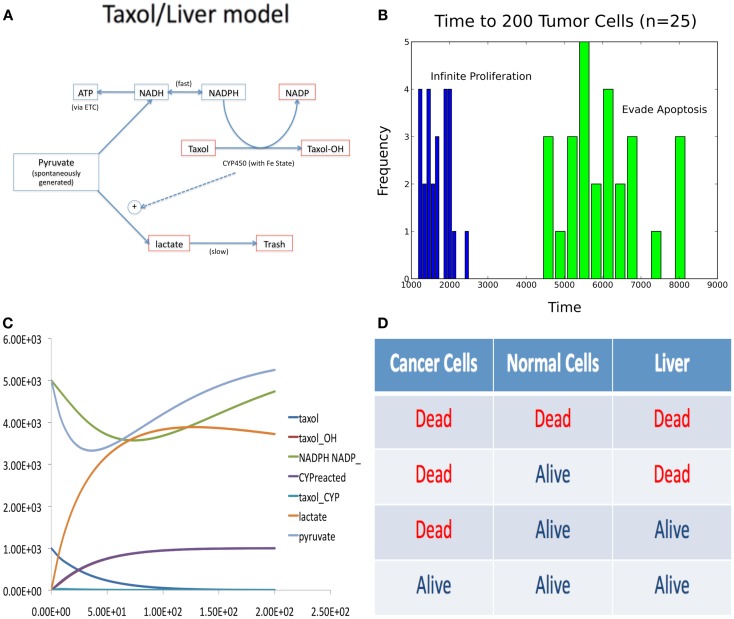
**Figures accompanying the composition model**. **(A)** Wiring diagram of liver model. **(B)** Time to tumor for different phenotypic aberrations. **(C)** Sample trace of ODE liver model simulation. **(D)** Four possible steady-state outcomes of composite model simulation, with different Taxol delivery schedules.

The problem was to model both liver toxicity in the presence of Taxol and also a population of cells in homeostasis (e.g., a tumor in a specific “cancer hallmark” state). Since the systems are not independent and are modeled with entirely different techniques, their simultaneous simulation is non-trivial. The liver model was constructed from the literature (Holmes et al., 1991; Rahman et al., 1994; Tamura et al., 1995; Manzano et al., 1996; Guengerich and Johnson, 1997) as a system of ODEs (depicted in Figure 4A) and the population as an agent based system, in which the cells signal one another to commit apoptosis or divide, determined by the population size.

The composition consisted of a KMC-like (kinetic Monte Carlo) simulation algorithm, in which the population model took discrete steps and the liver model was simulated continuously between the steps. Both models shared a common variable, tracking the concentration of Taxol in the organism. At the end of each model’s simulation “step,” the model continuously updated the global concentration of Taxol.

Deregulated growth in the population model was simulated by allowing one cell to either adopt a strategy of constitutive proliferation or evasion of apoptosis, which was then passed on to its offspring. The time it took for the mutant cell to produce 200 offspring is summarized in Figure 4B. Taxol is modeled as a diffusing agent that kills a cell when it tries to proliferate, thus targeting both mutant and wild type cells.

In the liver model, Taxol is metabolized and causes the build up of lactate, the main source of Taxol based liver toxicity. A sample trace of this metabolism is depicted in Figure 4C. The four possible effects of different dosing schedules for Taxol are depicted in Figure 4D. We discovered that it was possible to produce the optimal (3rd) effect in this model.

### 3.3 Next generation of dynamic models

To receive the best treatment and diagnosis, most cancer patients are willing to undergo invasive procedures to sample tumor and metastatic tissue. It will soon be possible to augment the data from these traditional means with non-invasively collected high-coverage DNA and RNA sequencing data, coupled to single-molecule and single-cell analysis with increasingly finer temporal granularity, using next-generation sequencing (NGS) technologies (Wigler, 2012). With such tools, we can study the diversity of individual cells in populations of cells.

Temporal models of dynamic biological processes, multi-scale and multi-level abstractions, and the analytical tools based on statistics and temporal logic could provide much sharper tools to address the challenges that these data pose.

A powerful approach is to build statistical analysis tools upon simple phenomenological models – in which the data themselves are viewed dynamically in terms of “snapshots in the temporal chain of events,” each event coordinated collectively by different cell-types in different cell-states (Ramakrishnan et al., 2010). With these logical analyses, inferred temporal logic invariants reveal various causal linkages between events that were earlier indistinguishable from mere correlations – recorded by the data and redescribed by the phenomenological models (Kleinberg and Mishra, 2009). The next steps in system biology’s progress in the biomedical arena would be improving our current understanding of mechanisms described by pathways, metabolic processes, signaling, etc., and in seeking to intervene in the components of these mechanisms to modify the system’s behavior (Olde Loohuis et al., 2014).

Success of such a program hinges on how we address the following questions (many of them partially solved):

(1) When can two models be considered “the same?”
(2) When can one model be considered an abstraction of another?
(3) When can one model be considered to approximate another model?
(4) How can several models be combined to provide larger models, either containing multiple subsystems or at multiple scales?

### 3.4 Multi-scale models

Computer science research has addressed several of these questions. Model equivalence provides tools such as simulation and bisimulation for defining and algorithmically testing whether two models represent the same trajectories of events. Model approximation extends these tools by allowing essentially equivalent models to be slightly different due to stochasticity or granularity. Model composition provides tools for combining disparate models both accurately and efficiently, by considering the models’ relevant interactions and independencies, respectively. This is tightly related to hierarchy and decomposition, which provide structures to efficiently represent, store, and execute composite models. Finally, evolution, while not inherently a computer science concept, is essential to understanding and modeling population effects.

Section 3.2 provides as example of a multi-scale, composite model of a tumor cell population, liver metabolism, and the simultaneous effects of Taxol treatment on both. Conceptually, this example helps illustrate the notion of combining two disparate types of models to study the emergent properties of the larger system. Practically, this model can serve as the basis to study the effects of various chemo-therapeutic dosage regimens, such as metronomic therapy, on the tumor and other organ systems.

### 3.5 Review of next-generation tools

We take the time here to illustrate hypothetical sequences of abstractions and to describe the types of tools that will be necessary in analyzing large scale dynamical models in modern systems biology.

#### 3.5.1 Illustration of model abstractions

Systems biology aims to describe large systems instead of isolated parts. It would be impractical to attempt to attain this goal with one model type, because different types of models lend themselves to modeling different types of systems, at different scales. To illustrate how the proposed approach permits a variety of modeling techniques to be applied to a single problem, we use a sequence of abstractions in which we can view the same system in many different ways. We start with a rule-based specification of a reactive biochemical system, which can be executed in a variety of ways.

**Figure 5:**
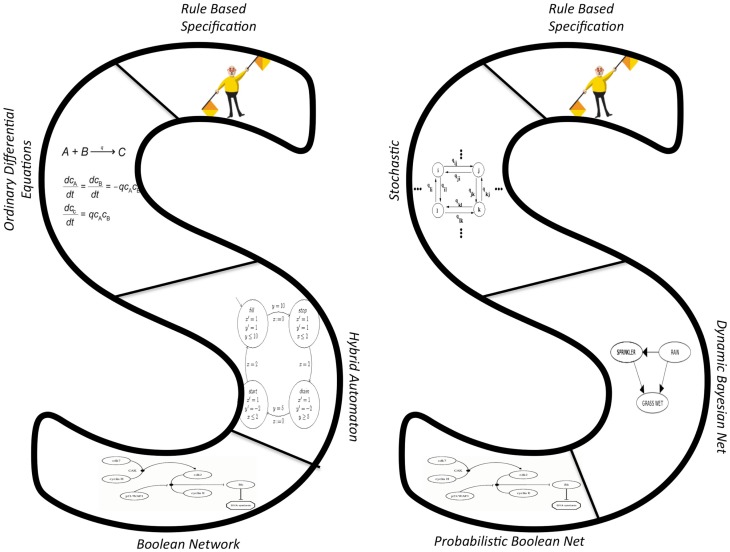
**Sequences of abstractions from rule-based specifications to deterministic (right) and stochastic (left) models**.

For instance, the specification can be transformed into an executable model that is either deterministic or probabilistic, as illustrated by the left (deterministic) and right (stochastic) sides of Figure 5.

First, in order to model the system using the sequence of deterministic models on the left-hand side of Figure 5, we start by assuming mass action kinetics. This permits tracking the average behavior of the chemical species using an ordinary differential equations model (see Section 2.1.2 for an example). This conversion is standard and well documented for rule-based models (Blinov et al., 2004; Danos et al., 2010). If the ODE dynamics exhibit sharp transitions among several regimes, each of which can be described by simpler ODE models, we abstract the ODE model into a hybrid automaton (HA). The HA contains discrete modes, each of whose dynamics is modeled by a simpler ODE model. This transformation has been defined and used in Alfieri et al. (2011), Grosu et al. (2011), and Noel et al. (2011). Alternatively, the ODE dynamics may be very steep. That is, molecular concentrations are either high or low but do not dwell in the intermediate states for long. In this case, the ODE model can be transformed into a Boolean network, in which there are activating edges from *x*_1_ to *x*_2_ if $\frac{dx_{2}}{dt}$ is positively related to the concentration of *x*_1_ and inhibitory edges if it is negatively related.

Next, consider the probabilistic side of the figure. Again starting with a rule-based model, it is appropriate to use a probabilistic model if the concentrations of species are low and stochastic effects could have significant effects on the overall dynamics. In this case, we transform the rule-based model into a stochastic model that simulates sampling from the chemical master equation (Danos et al., 2007; Smith et al., 2012) through a set of reactions and reaction rates.

Under the assumptions of a well-mixed and homogeneous system, this model can be simulated as a CTMC using the kinetic Monte Carlo (KMC) algorithm. To improve efficiency, at some cost to accuracy, we can transform this stochastic model into a dynamic Bayesian network (DBN). Through careful sampling, we can then find the distribution of reagent concentrations varying over time that is formalized by the DBN. If we further find that variable values tend to vacillate between a range of high and low values, we can model the DBN as a probabilistic Boolean network (PBN). Lähdesmäki et al. (2006) have explored the relationship between these two models and showed how they can represent similar systems.

#### 3.5.2 Tools description

##### 3.5.2.1 Model equivalence

When can we consider two models to be the same, so that we can justify substituting one kind of models by another? In what sense are they to be considered equivalent? What does this mean if models are stochastic – do they produce just the same aggregate results, such as averages, or must distributions be the same?

**Figure 6:**
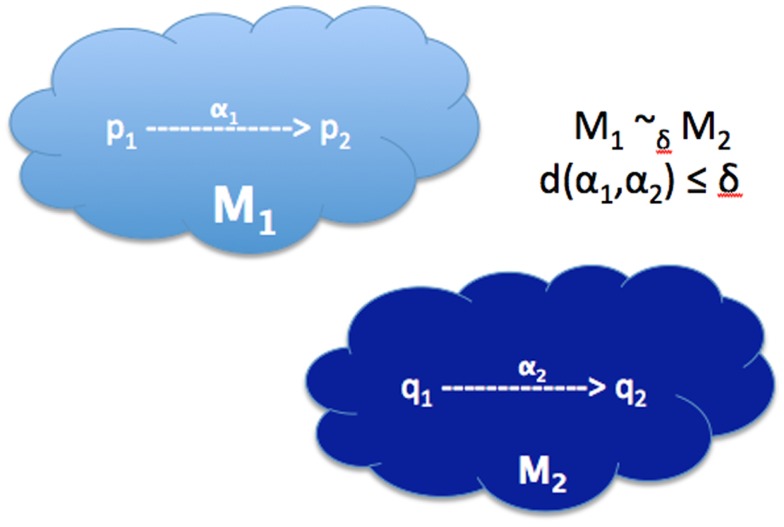
**Approximate bisimulation equivalence**. α_1_ and α_2_ are trajectories of simulations in $M_{1}$ and $M_{2}$, respectively. d(α_1_, α_2_) is the distance metric between the trajectories.

A very powerful concept for deterministic models is that of bisimulation (Desharnais et al., 2004; Danos et al., 2006), which was first developed in the context of reasoning about complex computational systems, such as an operating system. A bisimulation defines an equivalence between two models in terms of the simulation events (see Figure 6). Two models are thus equivalent if they can exhibit identical sequences of events for all possible simulations.

These ideas are usable even when the two models are of disparate types. To make this precise, define a trajectory as a set of states/observations produced by the simulation of a model. Two models ($M_{1}$ and $M_{2}$) are bisimilar ($M_{1}∼M_{2}$), if for every simulation in $M_{1}$, there is a simulation in $M_{2}$ that produces an equivalent trajectory, and vice versa. For this situation, a notion of bisimulation is required that can be used to ask if a model of apoptosis in one organism may be bisimilar to an analogous model in another organisms, even though the states in the two distinct organisms are described in terms of behavior of two different sets of genes, related by gene-orthology.

##### 3.5.2.2 Model approximations

Bisimulation equivalence is often too strong a constraint, and often approximate bisimulation equivalence (ABE) is sufficient for applications (Girard and Pappas, 2005). In ABE, we assume that the simulation trajectories for both models $M_{1}$ and $M_{2}$ lie in a single metric space (*X, d*). The models $M_{1}$ and $M_{2}$ are said to be approximately bisimulation equivalent up to precision δ if the corresponding simulation outputs are individually separated by distance at most δ. In this case, we write $M_{1}{∼}_{\delta }M_{2}.$

##### 3.5.2.3 Model compositions

Given a pair of models of interacting systems, we may wish to create a model that captures the essence of the combined system. Although, intuitively this is a rather simple concept, a good formal definition is difficult, as state-reachability and temporal dynamics interact in a complex way. One approach that has been used works by first defining a composition operation using a suitable heuristic and then showing that the resulting model is a “good” approximation of the real system. In a typical definition, the state of the composite model is described by a combination of the variables in its children’s states. If these variables do not overlap, the simulation of the composite model is trivial: the sub-models run in parallel, and the composite is their Cartesian product. When they share variables (e.g., crosstalk in a signaling network), parallel simulation may fail, as the flow of one may depend on variables in the other. A naïve approach would simulate both for ε time, implicitly assuming that the variables change only infinitesimally, update the flows of both, and repeat – which, however, is infeasible for continuous flows, as ε would have to approach 0 for accurate results; discrete flows are less problematic.

Consider three types of dependencies between a variable and a flow in different models.

- A close interaction: a small change in the variable causes a significant change in the flow.
- A remote interaction: a large change in the variable is required to cause a significant change in the flow.
- No (empty) interaction: no amount of change in the variable will affect the flow.

Clearly, no interactions would result in the trivial composition. The presence of one close interaction creates the “ε dilemma” discussed above. Thus, any partition that introduces such “ε dilemmas” is to be minimized. On the other hand, if all interactions between models were remote, we could define a guard condition for each interaction that is triggered when a variable changes sufficiently to require an update in its corresponding flow. The guard conditions constitute a set of discrete, timed events that are typically simulated using kinetic Monte Carlo.

##### 3.5.2.4 Hierarchy and decomposition

**Figure 7:**
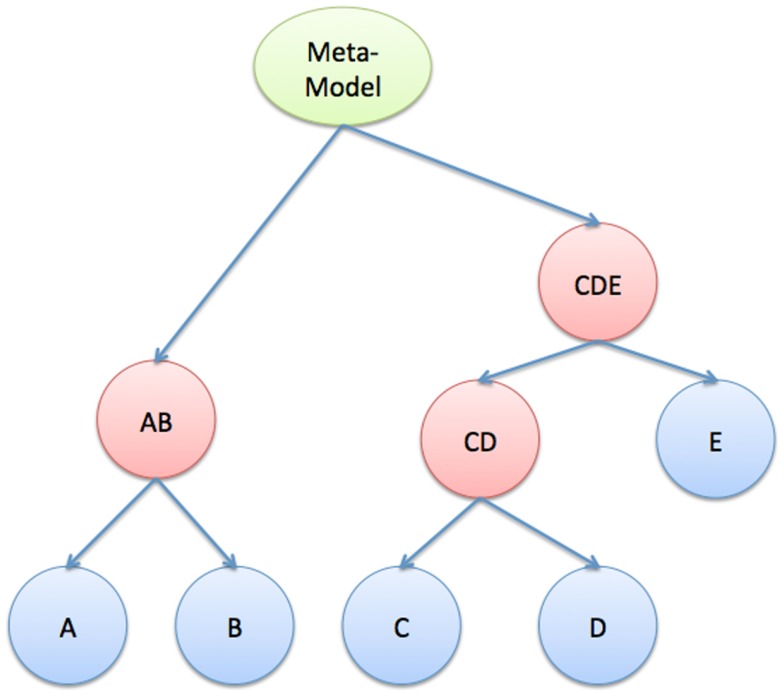
**Hierarchical composition**. Tree structure that describes the hierarchical relationships between atomic models, the meta-model, and partial-composition models.

We envision a large systems biology model as a hierarchical combination of smaller models. Thus, one can formulate the hierarchy as a tree structure (see Figure 7). The leaves (blue) represent atomic models that are well-defined outside of the compositional framework. The root (green) represents the full meta-model, and the other internal nodes (red) are partial-compositions of other models. Each node in this tree represents a complete executable model, defined by a state and a flow (see Section 2.2).

Clearly, to ensure that we can efficiently and accurately simulate such a large systems biology model, it is often required that it has a modular structure (Figure 7), in which intra-modular dynamics can be of types I, II, or III, but inter-modular dynamics can only be of type II and III. This requirement is not as stringent as it may seem at first; multi-cellular biological systems are naturally organized in this way. Intracellular dynamics are separated from one another by cell membranes but connected via slower acting, intercellular signals. Solid organs have their own internal dynamics and share “variables” via hormones and neuro-transmitters. Even gene and protein interactions in regulatory and metabolic systems can be decomposed into pathways that interact with each other through weak cross-talks (see Figure 1 for an example of crosstalk interaction between pathways).

To this end, we propose not only a formal structure in which to specify and simulate multi-scale models in systems biology but also a philosophy of modularity that follows the structures established by nature.

##### 3.5.2.5 Evolution

While “proximate” explanations in biology can be presented using mechanistic models of the kind we have described earlier, “ultimate” explanations are impossible except in the light of evolution, where the dynamics is to be understood in terms of multiple strategic agents. One powerful use of abstraction – built from approximations and compositions – is in allowing a translation from mechanistic models, in which the internal state is described in great details, to strategic models, in which the input and output behavior is characterized in terms of some less detailed internal states (phenomenological states). This shift allows us to connect mechanistic models to a burgeoning class of systems biology models that are based on game theory.

## 4 Discussion

From a pragmatic perspective, the study of cancer should aim to exploit patient data at all levels in drug discovery and therapy design. Analysis of data in the quantity currently available, with granularity at the level of a specific cell, requires more refined techniques than have been previously available. However, recent developments in modeling suggest that systems biology is primed to take the lead in this investigation, which necessitates the incorporation of large amounts of data into integrated models of multiple simultaneous processes operating at different scales.

Specifically, therapy design requires accurate, tractable progression models that track the evolution of pathway activity and genomic alterations that characterize various stages of the disease over time. To this end, we need rigorous notions of abstraction that allow us to retain detailed pathway information in simpler models. Therapy design must also take into account the toxicity of chemotherapy and budget constraints (e.g., the ones imposed by the monetary cost incurred by the healthcare system). Our approach requires integration among highly disparate models, and to this end, we need a rigorous way to simulate models simultaneously at different scales.

Finally, modern analytical tools will play a crucial role in the construction and application of these abstractions, hierarchical composite models. For instance, we need model checking to systematically characterize cancer phenotypes in terms of temporal properties. Also, sensitivity analysis is indispensable for identifying the key targets of signaling networks for drug discovery.

In summary, we need ways to simulate and analyze models efficiently. We also need to formalize model abstraction and to characterize its properties. These problems have been studied extensively in computational research, such as rate-distortion theory and bisimulation equivalence, and could now meaningfully be adapted to meet the needs of biological systems. Most importantly, we need a means to personalize complex heterogeneous models to patients, in order to devise the most effective therapies for each patient.

## Conflict of Interest Statement

The authors declare that the research was conducted in the absence of any commercial or financial relationships that could be construed as a potential conflict of interest.

## Footnotes

^1^Namely, mean and variance were used to approximate the distribution of transition time.
